# Abundance, survival, recruitment and effectiveness of sterilization of free-roaming dogs: A capture and recapture study in Brazil

**DOI:** 10.1371/journal.pone.0187233

**Published:** 2017-11-01

**Authors:** Vinícius Silva Belo, Claudio José Struchiner, Guilherme Loureiro Werneck, Rafael Gonçalves Teixeira Neto, Gabriel Barbosa Tonelli, Clóvis Gomes de Carvalho Júnior, Renata Aparecida Nascimento Ribeiro, Eduardo Sérgio da Silva

**Affiliations:** 1 Campus Centro-Oeste Dona Lindu, Universidade Federal de São João del Rei, Divinópolis, Minas Gerais, Brazil; 2 Departamento de Endemias Samuel Pessoa, Fundação Oswaldo Cruz, Rio de Janeiro, RJ, Brazil; 3 Departamento de Epidemiologia—Instituto de Medicina Social, Universidade do Estado do Rio de Janeiro, Rio de Janeiro, Brazil; 4 Centro de Pesquisas René Rachou, Fundação Oswaldo Cruz, Belo Horizonte, MG, Brazil; Faculty of Animal Sciences and Food Engineering, University of São Paulo, BRAZIL

## Abstract

The existence of free-roaming dogs raises important issues in animal welfare and in public health. A proper understanding of these animals’ ecology is useful as a necessary input to plan strategies to control these populations. The present study addresses the population dynamics and the effectiveness of the sterilization of unrestricted dogs using capture and recapture procedures suitable for open animal populations. Every two months, over a period of 14 months, we captured, tagged, released and recaptured dogs in two regions in a city in the southeast region of Brazil. In one of these regions the animals were also sterilized. Both regions had similar social, environmental and demographic features. We estimated the presence of 148 females and 227 males during the period of study. The average dog:man ratio was 1 dog for each 42 and 51 human beings, in the areas without and with sterilization, respectively. The animal population size increased in both regions, due mainly to the abandonment of domestic dogs. Mortality rate decreased throughout the study period. Survival probabilities did not differ between genders, but males entered the population in higher numbers. There were no differences in abundance, survival and recruitment between the regions, indicating that sterilization did not affect the population dynamics. Our findings indicate that the observed animal dynamics were influenced by density-independent factors, and that sterilization might not be a viable and effective strategy in regions where availability of resources is low and animal abandonment rates are high. Furthermore, the high demographic turnover rates observed render the canine free-roaming population younger, thus more susceptible to diseases, especially to rabies and leishmaniasis. We conclude by stressing the importance of implementing educational programs to promote responsible animal ownership and effective strategies against abandonment practices.

## Introduction

The relationship between dogs (*Canis familiaris*) and men goes back to the beginning of civilization, about 13,000 years ago [1, 2]. It is generally accepted that dogs were domesticated from the wolf (*Canis lupus pallipes* or *C*. *lupus variabilis*) in a process of symbiosis that evolved through selective breeding [3]. Indeed, dogs are termed as ‘domestic’ or ‘domesticated’ animals due to its association with humans and to the role that humans exercised in the emergence of this lineage [4]. Since domestication, this relationship became even more intense and dogs are ubiquitous in the cultural context of every society, constituting the most abundant carnivore animal on the planet [5]. Dogs have been associated with their owners’ welfare and well-being [6, 7] and have started to play different functions [2] due to their malleable personalities, docile behavior and utility as guardians and hunters [8].

Dogs that are not under immediate human supervision and have unrestricted access to public property are named “free-roaming” or free-ranging [1]. These terms encompass both owned dogs (family and some neighborhood dogs) and ownerless dogs (stray or feral) [3]. The existence of these dogs that can circulate freely in the streets can be harmful to both the animals and to human beings [1].

The abandonment and breeding of dogs in unrestricted environments have been attributed to behavioral, religious, cultural, ecological and socioeconomic factors, constituting important issues in public health and animal welfare [9, 10]. Unrestricted dogs, in general, have their psychological and physical health compromised, are more likely to acquire infectious diseases and have a lower life expectancy compared to pet dogs [11–13]. Their presence can be detrimental to humans since they are associated with the occurrence of biting incidents, transmission of diseases, damage to wild animal populations, accidents and pollution [14–19].

Different strategies are used to control the population of unrestricted dogs [20]. Elimination by killing is not considered effective, since the number of removed animals is compensated for the increased entry and survival of the remaining ones. In addition, this method is the subject of much criticism based on ethical issues [20–22]. As a result, actions towards promoting responsible animal ownership, the strengthening of legislation against abandonment, and surgical control have been established in different countries [20, 23]. Annually, thousands of unrestricted dogs are sterilized in veterinary clinics and in campaigns run by governments and non-governmental organizations. Nevertheless, the effectiveness of this measure, in the long term, has been poorly evaluated [24, 25]

A proper evaluation of actions that aim at controlling the free-roaming canine population requires non-biased estimates of parameters driving the dynamics of the target population [26, 27]. Even though several studies have yielded estimates of the population size of free-ranging dogs, most of them used inadequate analytical methods and were susceptible to biases, which casts doubt on the validity of these estimates, as evidenced in a recent systematic review [28]. Additionally, there is no published data of capture and recapture procedures that consider the canine populations as open, that is, subjected to deaths, births and migrations [29]. Despite the perceived need and usefulness of such parameter estimates and recommendations for the most appropriate approaches applicable under such study designs [30], survival and recruitment estimates of free-ranging dogs had not been obtained using methods of capture and recapture.

In this study, we present estimates of abundance, survival and recruitment rates, and the probabilities of capture of two free-roaming dog populations by means of analytical models for open populations, so far unexplored in previous studies. These dogs were followed for 14 months in a city located in the southeast region of Brazil. We report temporal variations of the estimates during the study period regarding gender and the effectiveness of surgical sterilization.

## Methods

### Study setting

The study took place in the municipality of Divinópolis, Minas Gerais, Brazil, the largest in the Central-West region of the state, with 228,643 inhabitants and a literacy rate of 94.7% (Brazilian Institute of Geography and Statistics–IBGE).

Regular public health measures involving dogs and implemented by the public services included sterilization of pet animals, when requested by owners, the capture and euthanasia of roaming dogs considered aggressive or diseased, and the capture of females along with their offspring for potential adoption by the local residents. Dogs not adopted were returned to the streets after the lactation period. Additional activities recommended by the Brazilian program on visceral leishmaniasis control, such as the culling of *Leishmania*-infected dogs, were also carried out on a regular basis [31, 32], as well as vaccination campaigns against rabies [33].

**Fig 1:**
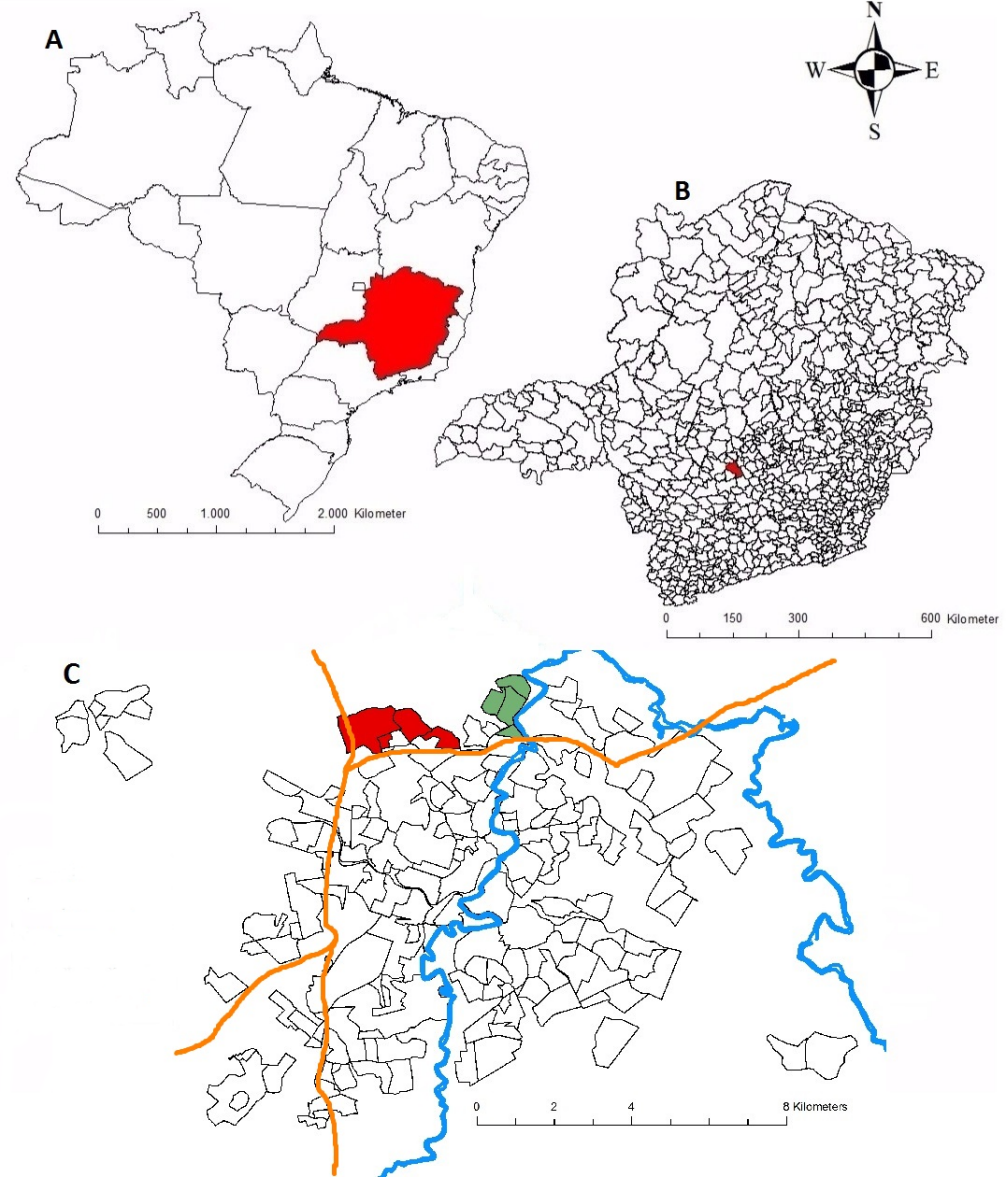
Study setting: a. Brazil, the State of Minas Gerais appears highlighted; b. the State of Minas Gerais, the city of Divinópolis appears highlighted; c. Divinópolis, the two areas of capture appear highlighted (area A–control—in red; area B–intervention—in green); River in blue; Highway in orange.

We selected two similar geographic areas (Fig 1): A (control) and B (intervention), with 3670 and 3900 inhabitants, respectively, according to the city hall registry. Both areas comprised four neighborhoods each and had similar profiles regarding size, household numbers and socioeconomic conditions.

### Ethical questions

Data collection and analysis complied with the ethical principles in animal experimentation as recommended by the Brazilian College of Animal Experimentation (COBEA). This project has been approved by CEPEA–Commission of Ethics in Research Involving Animals of the Federal University of São João del Rey (protocol no. 24/2010).

### Pilot study

Prior to the implementation of the activities described in the next section, we performed a pilot study to define the areas of study and to identify potential problems requiring further attention. The pilot study lasted for four days, each day spent in a different neighborhood of the city. Each one of the four neighborhoods belonged to four different eligible areas defined with municipal health authorities based on previous information on the occurrence of free-roaming dogs and on the feasibility of carrying out the research. Those with similar features and with the highest raw number of captured and released animals were selected. Data acquired in this stage were not used in the analyses.

### Captures and recaptures, identification and intervention procedures

We conducted seven capture and recapture procedures in a period of one year and two months, one every two months. Dogs found wandering in the streets during the capture period were included in the study, provided an owner with a dog leash did not accompany them. Adapted vehicles drove around all the streets of the study areas screening for free-roaming animals. The work team consisted of one driver, two municipal health agents, one veterinarian and two individuals responsible for collecting and recording data. In Area A, activities took place in the first week of the collecting months, while in Area B, they took place in the second week of the same month. Screenings always followed the same route, so that they covered all the streets of each region at least once. The same team collected the data in both areas.

Captured dogs of areas A and B were taken to the Health Surveillance Reference Center of the city (CREVISA) in adapted vehicles of Public Health Service. In CREVISA they underwent clinical tests conducted by veterinarians and were screened for canine leishmaniasis (CanL). The diagnosis of CanL was made in the Parasitology Laboratory of Universidade Federal de São João del Rei through the techniques of “Enzyme-Linked Immunosorbent Assay” and “Indirect Fluorescent Antibody Test”. Seropositive dogs were euthanized according to the recommendations of the Brazilian Ministry of Health [31]. Dogs that tested negative were microchipped for identification if recaptured. These animals were also de-wormed and received a vaccine against rabies and another against distemper, leptospirosis, hepatitis, parainfluenza, parvovirus, coronavirus and adenovirus. Dogs captured in the Area B (intervention) were sterilized (S1 Appendix). Healthy animals were returned to the same place where they had been captured, after screening for CanL and total rehabilitation of the surgical procedure (Area B dogs) (S1 Appendix).

Recaptured animals were re-examined. Animals screened and found negative for CanL were released again. Animals that tested positive were euthanized. All dogs, even those not captured, were photographed for posterior identification in the same sample interval and in recaptures. For identification, distinctive characteristics of the dogs were sought in their craniolateral and/or dorso-caudal portions. For analytical purposes, dogs not physically captured but photographed were considered captured. This information was added to the database serving as input for the estimation of the population dynamic parameters.

We released informative materials to the local population with the purpose of increasing their awareness regarding responsible animal ownership and visceral leishmaniasis (S2 Appendix).

### Data registry

We entered the individual history of capture and recapture of each animal into a Microsoft Excel (2013) database formatted as “encounter history” for captured animals tagged alive [30]. Euthanized animals carried a negative sign, indicating the occurrence of death during the capture procedure. There were no deaths attributed to other factors.

### Analysis

#### General procedure

The Jolly-Seber model with POPAN parametrization served as the starting structure for model fitting [34]. We estimated the following three parameters using his approach:

φ_i_ (survival): probability of a marked or unmarked animal surviving (and not migrating) between the captures i and i+1.
p_i_: (capture probability): the probability of finding or seeing a marked or unmarked animal in a given capture i, given the animal is alive and in the area of capture.
b_i_: (probability of entrance): considering the existence of a “super-population”, comprised of all animals that would ever be born to the population, this parameter constitutes the probability of an animal of this hypothetical “super-population” entering the population between the occasions i and i+1.

The parameters above allow for the estimation of Recruitment (B: number of animals that enter the population between two capture procedures) and population size (N).

We used Mark, version 6.2 for fitting the statistical models.

#### Goodness of fit of highly parameterized models

We evaluated the goodness of fit (GOF) of the model with the largest number of parameters prior to fitting models that were more parsimonious [35]. This step was necessary to check the premises of the Jolly-Seber approach. We checked model’s GOF based on tests 2 and 3 of the Release suite of Mark software, the GOF statistics obtained via the bootstrap, as well as the “median c-hat” statistics. Among the procedures, we adopted the one that indicated the highest variance inflation factor (c-hat).

We first considered the model with the variables “sex” (male or female), “area” (A or B), “time” (sampling period), and their respective interaction terms. A c-hat value of 2.52 suggested sparsity in different periods of capture. We changed our model search strategy accordingly and partitioned the previous model into two models: a model with “sex”, “time” and interactions and a second model with “area”, “time” and interactions. C-hat estimated in these cases was 1.17 and 1.25, respectively for each model, and there were few indications of sparse data.

#### Modeling procedures

The GOF analysis reported above prompted us to investigate factors associated with survival estimates, probability of capture and probability of entry separately for the variables “sex” and “area”. In both cases, we built models considering time-dependent or time-independent parameters and the presence of interactions between the variables “sex” and “time” or “area” and “time". We also fitted additive models containing parameters expressed as a function of two or more factors, in this case, area and time or sex and time, without the presence of interactions. In total, we fitted 50 models in both groups (S3 Appendix). All models supported temporal variations for the “probability of entry” (b_i_).

Model selection followed the usual approach by searching for the most parsimonious structure that retained the best balance between explained variability and precision of estimates. We ranked all models based on Akaike’s Information Criterion corrected for finite sample sizes (AICc). This statistic provides a summary balance between the goodness of fit to the data of each model and the number of necessary parameters. “Data cloning” was used to identify the correct number of estimated parameters [36]. The presence of overdispersion in the data indicated the need to further correct the AICc statistics by the C-hat values to obtain the quasi-AICc statistics (QAICc). Lower values of these latter statistics point to models that were more parsimonious [37].

After ranking all models based on QAICc, we evaluated the force of evidence in favor of each model (AIC weight–“w”). This statistic can be interpreted as the conditional probability of a given model being the best among the set analyzed. Thus, higher values of “w” indicate higher force of evidence in favor of the model. Models with values of “w” lower than 0.01 were disregarded.

We further evaluated the importance of each variable in a context of a set of models by adding up the weight (w) of each model containing a given variable [37]. We repeated this procedure for all predictors considered. Variables with higher weights are considered more important than those with lower weights in explaining the variance observed in the data.

#### Parameter estimation

Estimates for the parameters survival probability, probability of capture, probability of entry in the population, abundance and recruitment rate relied on the technique known as “model averaging” [38]. Under this approach, we calculated the weighted average of parameter estimates from all models fitted to data using as weights the relative support (w) of the respective model. Therefore, this technique accounts for both sources of variance: the specific conditional variation present in each one of the models and the nonconditional variation present in the model selection process. In this way, parameter estimates express more faithfully the sources of uncertainty associated with the estimation process.

In time-dependent models under POPAN not all parameters are identifiable [30]. This is the case of the probability of capture in the first and in the last captures (p_1_ and p_k_), the probability of entry between the first and the second captures (b_1_) and between the penultimate and the last captures (b_k-1_), and the survival probability between the penultimate and the last captures (φ_K-1_). Thus, only the remaining parameters whose estimation was possible are described here.

The effectiveness of sterilization was analyzed by comparing the evolution of abundance and of the other parameters estimated in the areas: A (control) and B (intervention).

We estimated dog:human ratio by the ratio of population size and the dog mean abundance in each of the areas.

## Results

### General description

During the study period, 171 dogs were identified individually in region A (control) and 157 in region B (intervention). The proportion of males in areas A and B was 56% (96 dogs) and 62% (98 dogs) respectively. One hundred and thirty-three animals (77 males and 56 females) were captured in more than one effort of captures. One hundred and thirty-eight dogs (88%) were sterilized. Twenty-four were euthanized for testing positive to CanL.

**Fig 2:**
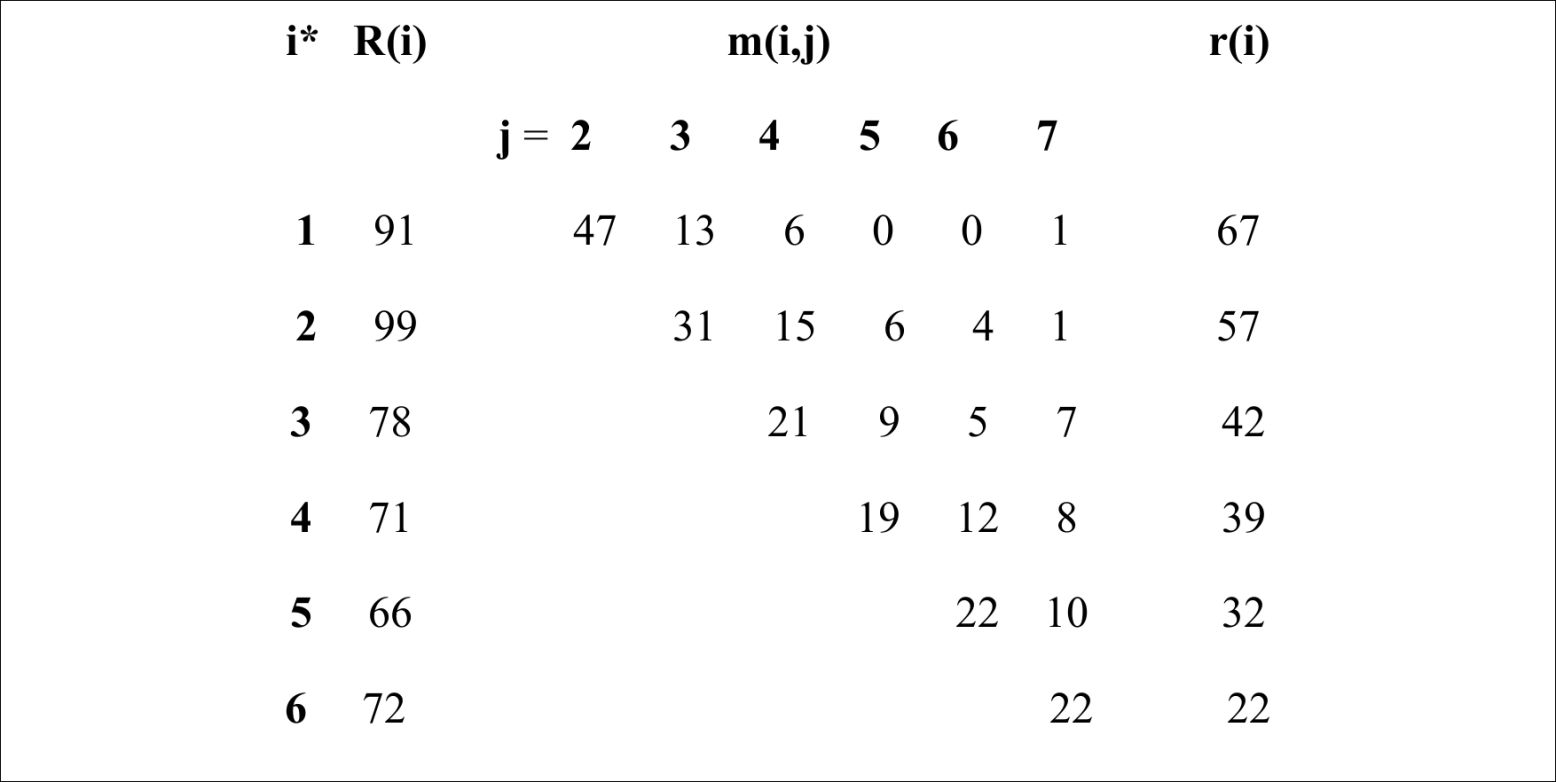
Number of dogs released in each capture and recaptured in the subsequent efforts of captures. *i = capture effort; R = number of animals released alive just after a capture; r = total of animals released and recaptured at least once; mij: number of animals recaptured for the first time subsequent to a specific effort i.

Sixty-six different individual histories of captures were registered and 38 of them included animals not captured in the first effort. All recaptures and visualizations took place in the same area where the dogs were initially detected. Most free-roaming dogs were neighborhood dogs, i.e. several human residents in the area provided the needed resources to them [3]. Fig 2 describes the number of released dogs in each capture and recapture in the subsequent efforts of captures. S4 Appendix presents individual histories, stratified by sex and area.

### Selection of models and force of variables

#### Models including the variable “gender”

We fitted 50 models containing the variable gender to the data (S3 Appendix). Five had w-statistics greater than 1% and are shown in Table 1, along with statistics QAICc, ΔQAICc (difference, in module, between QAICc values of the best model and the analyzed model). The relative support for each model is also expressed as the ratio of its w-statistics to the largest value of this statistic among the five models considered.

**Table 1:** Most parsimonious models containing variable gender.

Model (i) #	QAICc	Δ QAICc	Weight (w)	Support (w_1_/w_i_)
**1. Φ**_**t**_ **p**_**t**_ **b**_**t**_	1129.1074	0.0000	0.73242	X
**2. Φ**_**g+t**_ **p**_**t**_ **b**_**t**_	1132.8450	3.7376	0.11302	6.48
**3. Φ**_**t**_ **p**_**g+t**_ **b**_**t**_	1133.3565	4.2491	0.08751	8.37
**4.Φ**_**g+t**_**p**_**g+t**_ **b**_**t**_	1134.7524	6.6450	0.04355	16.82
**5. Φ**_g*t_ **p**_**t**_ **b**_**t**_	1137.0836	7.9762	0.01358	53.93

#. Φ = survival; p = probability of capture; b = probability of entry; t = parameter values vary in different capture occasions; g+t = additive model in which parameter values varied with gender and time; g*t = interaction between gender and time.

The model in which survival, probability of capture and probability of entry varied with time, but not between male and female dogs, was considered the most parsimonious (w = 73.24%). The weight for this model was 6.48 times higher than the model in which survival varied additively with gender; and 8.37 times higher in relation to the model in which the probability of capture varied between genders. Other models had weights lower than 5% and low support, when compared to the most parsimonious model.

The sum of each variable’s weight (w) considering all models (Table 1) are presented in S5 Appendix. Time-dependent parameters displayed higher weights. Survival and capture probabilities varied between genders but these variables’ “w” conferred weak support to this statement. For entrance probability, there was no evidence for the existence of group variation.

#### Models with the variable “area”

Models containing the variable “area” behaved similarly to the previous set containing the variable “gender”. Table 2 presents the six models in this group with w-statistics greater than 1%. Results for the remaining models are described in S3 Appendix.

**Table 2:** Most parsimonious models containing the variable area in which the dog was captured.

Model (i) #	QAICc	Δ QAICc	Weight (w)	Support (w_1_/w_i_)
**1. Φ**_**t**_ **p**_**t**_ **b**_**t**_	1067.5657	0.0000	0.57923	X
**2. Φ**_**a+t**_ **p**_**t**_ **b**_**t**_	1069.8357	2.2700	0.18618	3.11
**3.Φ**_**a+t**_**p**_**a+t**_ **b**_**t**_	1070.3336	2.7679	0.14515	3.99
**4. Φ. p**_**a+t**_ **b**_**t**_	1073.3713	5.8056	0.03178	18.22
**5. Φ**_**t**_ **p**_a*t_ **b**_**t**_	1073.5471	9.9814	0.02911	19.90
**6. Φ**_a*t_ **p**_**t**_ **b**_**t**_	1075.4533	7.8876	0.01122	51.62

#. Φ = survival; p = capture probability; b = entry probability; t = parameter values vary in different capture events; a+t: additive model in which there is variation in the parameter values in area and time; a*t: interaction between area and time.

The model containing time-dependent parameters, but constant between areas control (A) and intervention (B), was the most parsimonious in this group. Its weight, however, was lower when compared to the models containing the variable “gender” (Table 1). It had 3.11 times more support from the data than the model in which there was variation in survival between areas, and 3.99 more support than the model in which the probability of capture also varied between areas. Differences were significantly higher when comparing the most parsimonious model to the remaining models since the latter models received even lower support from the data.

We observed stronger weights associated with time-dependent variables (S5 Appendix). However, the observed weights were lower than those associated to the variables in the set of models containing the variable “gender”. We also observed stronger weights in variables describing differences in survival and probabilities of capture between areas. The remaining variables were associated with lower weights. In particular, the probabilities of entry did not vary between control and intervention areas.

### Estimates

#### Models with the variable “gender”

We estimated a population abundance of 148 females and 227 males in the target population in the entire study period. Table 3 depicts gender-specific parameter estimates and their respective confidence intervals (CI). They result from model-specific estimates weighted by the relative support (w) of the respective model. Taken together, these results show that gender-specific differences regarding the estimated parameters were not relevant. In contrast, time-dependent differences were significant. Survival probabilities increased steadily, going from 0.75 in the interval between the first and second captures, to 0.99 between the fifth and sixth. On the other hand, probability of entry in the population was close to zero between the fifth and the sixth captures, and varied between 0.12 and 0.15 in other intervals. Probability of capture reached the highest value in the second capture (0.68), and decreased subsequently until the fifth capture when it reached its lowest value (0.39). Estimates of abundance highlight the majority of males in its composition. Additionally, there was a higher entry of male dogs in all intervals in which the number could be estimated. Population increased in size during the study. We estimated the presence of approximately 59 females and 92 males in the second capture, 71 and 69 females and 104 and 105 males, respectively, in the fifth and sixth captures.

**Table 3:** Estimates for sex.

Survival (CI)		Capture probability (CI)		Entrance probability (CI)		Recruitment (CI)		Abundance (CI)	
Female	Male	Female	Male	Female	Male	Female	Male	Female	Male
**1 = 0.75 (0.62–0.86)**	0.75 (0.61–0.85)	**1** = NI	NI	**1** = NI	NI	**1** = NI	NI	**1** = NI	NI
**2 = 0.80 (0.62–0.91)**	0.79 (0.62–0.91)	**2** = 0.68 (0.55–0.79)	0.68 (0.56–0.79)	**2** = 0.12 (0.06–0.22)	0.12 (0.06–0.22)	**2** = 15.40 (5.23–25.56)	23.64 (8.00–39.21)	**2** = 58.67 (48.14–69.18)	91.88 (75.58–108.17)
**3 = 0.77 (0.57–0.89)**	0.76 (0.57–0.89)	**3** = 0.51 (0.40–0.62)	0.51 (0.40–0.63)	**3** = 0.12 (0.05–0.24)	0.12 (0.05–0.24)	**3** = 15.30 (3.67–26.94)	23.60 (5.73–41.45)	**3** = 61.77 (49.65–73.89)	94.49 (75.87–113.11)
**4 = 0.84 (0.57–0.96)**	0.84 (0.56–0.96)	**4** = 0.48 (0.37–0.60)	0.48 (0.37–0.60)	**4** = 0.15 (0.08–0.24)	0.15 (0.08–0.24)	**4** = 19.13 (8.94–29.31)	29.44 (13.78–45.10)	**4** = 62.72 (49.64–75.80)	94.28 (74.35–114.21)
**5 = 0.99 (0.96–1.00)**	0.99 (0.99–1.00)	**5** = 0.39 (0.30–0.49)	0.39 (0.30–0.49)	**5** = 0.0001 (0.00–0.004)	0.0001 (0.00–0.004)	**5** = 0.00 (0.00–0.63)	0.01 (0.00–1.00)	**5** = 71.19 (59.00–83.36)	105.27 (82.25 - (123.30)
**6 = NI**	NI	**6** = 0.42 (0.32–0.52)	0.42 (0.32–0.52)	**6** = NI	NI	**6** = NI	NI	**6** = 69.16 (56.83–81.48)	104.27 (86.22–123.31)
		**7** = NI	NI					**7** = NI	NI

NI: Non-identifiable parameter; CI: Confidence interval

#### Models with the variable “area”

We estimated the presence of 199 dogs in area A (control) and 177 in area B (intervention) throughout the study period. Estimates of additional parameters stratified by control and intervention areas are presented in Table 4. They reflect the weighting mechanism by the relative support of all models analyzed as explained in the Methods section. Analogously to the models containing gender, differences between the estimates of each area were small, even though they were slightly higher than those seen between genders. Owing to the fact that stronger weights were attributed to models that did not show a difference between strata in either set of models, estimates of survival, capture probabilities and entry probabilities for the areas were similar to those described for gender. On the other hand, the recruitment was similar in both areas, contrasting with our findings comparing males and females. Population size increased in both areas. Abundances seen in the second capture were smaller, 82 animals in Area A and 70 in area B, contrasting with abundances observed in the fifth capture, 96 dogs in Area A and 83 in Area B.

**Table 4:** Estimates for area.

Survival (CI)		Capture probability (CI)		Entrance probability (CI)		Recruitment (CI)		Abundance (CI)	
Area A	Area B	Area A	Area B	Area A	Area B	Area A	Area B	Area A	Area B
**1 = 0.74 (0.59–0.85)**	0.76 (0.62–0.86)	**1** = NI	NI	**1** = NI	NI	**1** = NI	NI	**1** = NI	NI
**2 = 0.80 (0.61–0.91)**	0.81 (0.63–0.92)	**2** = 0.68 (0.54–0.79)	0.69 (0.56–0.80)	**2** = 0.12 (0.06–0.23)	0.12 (0.06–0.23)	**2** = 20.91 (6.17–35.66)	18.34 (5.45–31.23)	**2** = 81.74 (66.37–97.10)	**2** = 70.33 (57.13–83.52)
**3 = 0.76 (0.55–0.89)**	0.80 (0.58–0.90)	**3** = 0.49 (0.39–0.62)	0.51 (0.39–0.64)	**3** = 0.12 (0.05–0.25)	0.12 (0.05–0.25	**3** = 20.57 (3.41–37.72)	18.66 (3.60–32.71)	**3** = 84.76 (67.00–102.54)	**3** = 73.99 (59.11–88.88)
**4 = 0.84 (0.53–0.96)**	0.85 (0.56–0.96)	**4** = 0.47 (0.34–0.59)	0.48 (0.36–0.60)	**4** = 0.14 (0.08–0.25)	0.14 (0.07–0.25)	**4** = 25.40 (9.93–40.86)	21.64 (7.47–35.80)	**4** = 84.44 (65.00–103.88)	**4** = 75.56 (58.88–92.24)
**5 = 0.99 (0.91–1.00)**	0.99 (0.91–1.00)	**5** = 0.39 (0.29–0.49)	0.40 (0.30–0.51)	**5** = 0.01 (0.00–0.02)	0.01 (0.00–0.05)	**5** = 0.08 (0.00–1.00)	**5** = 1.00 (0.00–1.00)	**5** = 95.52 (76.63–114.41)	**5** = 82.64 (66.49–98.80)
**6 = NI**	NI	**6** = 0.41 (0.31–0.52)	0.43 (0.32–0.54)	**6** = NI	NI	**6** = NI	NI	**6** = 93.84 (74.60–113.08)	**6** = 80.85 (65.22–96.00
		**7** = NI	NI					**7** = NI	NI

NI: Non-identifiable parameter; CI: Confidence interval

Dog:human ratio in Area A was one dog to 42 human beings. In Area B, this ratio was one dog to 51 humans.

## Discussion

We estimated critical parameters (survival, recruitment and abundance) that describe the population dynamics of free-roaming dogs based on a capture and recapture study design and on models suitable for open populations. Our study demonstrated the increase in population size in both areas, the predominance and greater recruitment of males, the temporal variability in recruitment and in survival probabilities, the lack of effect of sterilization on population dynamics, the influence of abandon and of density-independent factors and a high demographic turnover. Such information on the dynamics of free-ranging dogs are useful for informing control interventions of unrestricted dog populations and against canine visceral leishmaniasis and rabies, both neglected tropical diseases endemic to various countries.

The dog:man ratio observed in our study was smaller than that observed in counts performed in urban regions of Nigeria (1 dog to 25 men) [39] and that among rural dog populations in India (1 dog to 35 men) analyzed by mean of Beck’s Method [40]. It was larger, however, than the counts obtained by Hossain et al. [41] in a rural area of Bangladesh. Demographic, socioeconomic, environmental and cultural factors able to explain differences in abundances between and within regions have been underexplored in the literature [28]. Abundance of free-roaming dogs in general is lower in rural than urban areas [42, 43]. Regions under poorer socioeconomic conditions and higher population densities tend to have a larger concentration of dogs [44]. In the present study, abundance possibly reflects the intermediary socioeconomic condition, the urban environment and low population density of the study areas as well as the different methodology applied.

For most animal species, survival is the demographic parameter with highest impact on population size [45]. Few studies, however, aimed at estimating the survival of free-ranging dogs in urban environment. Reece et al. [46] used data from a sterilization program to estimate the survival of castrated females in Jaipur, India. Annual survival of females aged over one year old was 0.70 and of females in their first year of life was 0.25. The assumptions leading to these estimates were implausible and might have biased the results. Pal [47] conducted four annual capture efforts, in Bengal, India, and estimated the canine mortality from the number of dogs observed in the captures after the first one. Annual survival for adult dogs was 0.91, and for dogs in their first year of life, 0.18. This study did not report capture probabilities and included in estimation only dogs found dead. This approach possibly contributed to an overestimation of the survival probability. Survival probability reported by Beck [1], in a study conducted in Baltimore, Canada, with dogs of all age groups, was 0.70. This author relied only on existing information regarding the number of dead dogs, also possibly leading to an underestimation of mortality and consequently to an overestimation of survival probability.

Although limited, estimates obtained in the literature suggest that survival is lower in young free-roaming dogs [46, 47], a pattern already seen in different animal species [48, 49]. Once the proper identification of the dogs’ ages was outside the scope of our project, estimates in the present study refer to the general survival probability of the population and not age-specific probabilities. Annual survival in our study was higher than that estimated for dogs aged less than one year [46, 47], and lower than the survival probability estimated for adult dogs [46, 47] and for Beck’s study population [1]. The low survival probability identified in the population results from the different sources of mortality experienced by free-roaming dogs in the study setting. Residents often reported roadkill and poisoning episodes during the study period. The high prevalence of CanL-seropositive dogs, especially in the first months of the study, is another relevant factor leading to the removal of many animals by euthanasia. Additionally, government actions towards street dogs were restricted to rabies vaccination. The lack of additional prophylactic measures or treatment may have contributed to the increased susceptibility of dogs to infections and other conditions.

Females have lower survival rates [50, 51] in a large number of animal species due primarily to the effects of reproduction. Given the predominance of males in different studies, it is hypothesized that this pattern also happens in the canine populations [52]. We observed no difference in survival probabilities between genders, although a higher abundance and recruitment of males occurred. Most pet owners prefer male dogs since they do not get pregnant and are better guard dogs [28, 41]. Therefore, the higher survival of male puppies of owned but free-ranging dogs or of pet dogs subsequently abandoned by their owners could probably explain the predominance of males in the free-roaming dog population.

To our knowledge, we report for the first-time the temporal evolution of the survival probability of free-roaming dogs. Annual point estimates of survival probability found in the literature do not bear a longitudinal structure. Our results show that survival of unrestricted dogs displays variations, even in short temporal scales. Among the models fitted to the data, those in which survival did not vary with time had significantly lower weights, indicating that a constant value is not appropriate to representing the entire period. Estimates of survival probabilities in other mammal species also show a temporal dependence, especially in young individuals [53–55]. Long-term studies are required to uncover the intrinsic and extrinsic determinants driving these temporal dependencies. This would be useful for understanding the population dynamics of free-ranging dogs and improving the validity and precision of predictive modelling procedures. Such studies are difficult to perform, and thus are rare in the literature [56]. Despite being a short-term study, survival, recruitment and population size displayed an increasing tendency. This pattern suggests that density-independent factors could be responsible for driving the variations observed in survival probabilities of dogs in both areas.

Density-dependent mechanisms are the subject of several studies focusing on different animal species [57–60]. In epidemiological and ecological modeling, one assumes that survival and recruitment rates in free-ranging dogs are driven by the availability of resources in the environment, a density-dependent mechanism [61]. However, as pointed out by de Little et al. [56], extrinsic factors not regulated by density may determine fluctuations in population size when those populations have not yet reached their carrying capacity or when environmental conditions are favorable. According to Morters [61], human beings are the major agents responsible for providing care and adequate food for dogs. As a result, human related factors such as living together with free-ranging dogs, the low dog-human ratio and the availability of residents’ resources to maintain these animals, may explain why the increase in density had no influence upon mortality and recruitment. Reducing the availability of shelters and food is an ethically questionable measure for population control of free-roaming dogs. However, this alternative has been presented in a recent study [62].

It is not possible to affirm whether population growth, attributed to the large number of animals entering the population, would keep the reported increasing trend constant if the study had a longer duration. Maximum survival and lack of recruitments between the fifth and the sixth captures suggest potential instabilities. In the presence of increasing abundance, density-dependent factors could start to play a stronger role in regulating the population [63, 64] and in the behavior of residents regarding their support to dogs. There is considerable uncertainty in assessing the role played by vital rates and intrinsic and extrinsic factors in driving the population size of free-ranging dogs and other mammals [54, 61, 65, 66].

Estimates of recruitment obtained from capture and recapture models do not allow us to disentangle the sources of entry attributable to births and immigration. We observed no females with their brood along the study period. We might infer that breeding females were located in less visible areas or put to adoption by the city public service and returned to the streets after the lactation period, even though such registries were rare. In the study of Morters et al. [61], as well as in the present study, recruitment was driven, predominantly, by the arrival of adult animals. The recruitment contingent may comprise dogs born in the region and not identified as puppies, dogs from other regions that migrated to the study region or were relocated by residents who raised them unrestrictedly, previously restricted dogs that changed status to being freely raised, or dogs abandoned nearby that later joined the population. The study areas are geographically isolated from their neighboring regions and are located next to a highway where dogs were frequently abandoned. Therefore, the latter mechanism seems more plausible to account for the increase in population size rather than the spontaneous immigration of dogs. Although there are heterogeneities [67], free-ranging dogs are territorial animals that, in general, do not move across long distances, unless forced by unfavorable environmental conditions [1]. The low mobility of dogs in a favorable environment is supported by our data, since there were no animal movements between the areas A and B.

The replacement of a great number of dogs that died or emigrated by dogs that are born or immigrate, as observed in our study populations, drives the population structure and gives rise to health problems that result from these structures. A population with a high turnover may be more susceptible to diseases [24]. A high population turnover is the major obstacle for the success of control strategies against rabies in developing countries [68]. Vaccination strategies under such population dynamics must occur in short intervals and achieve high coverage in order to maintain proper levels of immunization. On the other hand, the replacement of euthanized dogs by susceptible animals and new individuals entering the reservoir compartment are the main causes of the low effectiveness of the euthanasia of seropositive dogs, a control strategy adopted in Brazil against leishmaniasis [69]. In addition, the population also becomes younger and more likely to acquire other infections under the high turnover regime [70].

The field of mammal ecology identifies two main reproductive strategies driving population size, each focusing on specific stages of the life cycle. The so-called “slow breeding” animals experience late maturation and their reproductive strategy depends on the survival of juveniles and young adults. On the other hand, “fast breeding” mammals complete their reproductive cycle within their first year of life and place emphasis on fertility as their survival strategy as a species [71, 72]. Control of the population size of “fast breeding” animals, such as dogs [73], is more effectiveness when relying on measures that restrict entry of new individuals into the population as opposed to subjecting animals to euthanasia, a practice that reduces adults’ survival. The fast versus slow breeding rationale, the sensitive ethical issues, and the low effectiveness of euthanizing animals observed in regions where this practice has been applied [20, 61, 74] prompted us to only consider sterilization, and not culling, as an alternative control strategy in our study. Its use as a population control measure against hydatid disease in developing countries, however, has been recommended [19].

It is worth noting that in the present study sterilization did not affect the canine population dynamics. After one year and two months, we observed no difference in survival, entrance or recruitment probability between the control region and the intervention area where 88% of the dogs were sterilized. The impact of sterilization takes place slowly as suggested by modeling exercises. It might take up to five years for the first impact of sterilization to become apparent and up to 30 years of uninterrupted efforts to reach its maximum impact [75]. Reece and Chawla [24] evaluated a program that surgically sterilized 19,129 neighborhood dogs in Jaipur, India, for eight years and showed that the population declined by only 28 per cent. On the other hand, Frank and Carlisle-Frank [76] observed only a small impact of a sterilization program on the number of dogs joining a shelter in the United States. Amaku et al. [77], based on results from a mathematical model developed specifically for stray dogs, concluded that sterilization becomes inefficient in the presence of high abandonment rates, even after prolonged periods of use. Natoli et al. [78] reached the same conclusion after studying for 10 years the impact of a castration and devolution program on a non-restricted cat population. Continuous negligent practices of animal ownership, including abandonment, had a negative impact on the sterilization strategy rendering it ineffective and countering the effect of 8,000 surgical interventions undertaken in that study. Finally, in a study conducted in Brazil, Dias et al. [79] concluded that it is counter-productive to invest in sporadic sterilization campaigns of owned dogs, the currently strategy adopted in most of Brazilian municipalities.

The small impact in controlling the population size, especially in areas with high abandoning rates as in our case, the need to reach high coverage rates without interruptions, the absence of behavioural benefits for castrated dogs [80], high costs and null impact on a short-term perspective, minimize the relevance of sterilization of free-ranging dogs in managing the population and controlling diseases. In this context, it becomes apparent that public health services and non-governmental organizations must develop and prioritize more effective strategies against abandonment practices. In countries where free-ranging dogs are considered a humanitarian or a public health issue the implementation of educational programs addressing responsible animal owning at different levels, the registration of dogs and their owners and the improvement of legislation aimed at those who wish to have a pet becomes imperative [81].

Probability of capture is a useful parameter in the identification of essential population features [82]. It is known to vary in space, time and among individuals [83]. Although we observed no significant differences in this parameter between genders and areas, it varied over time even in the presence of standardized procedures. Such fluctuations may be attributable to social organization features not yet investigated in the population, or to environmental and climatic factors. Dias et al. [84] showed that weather exerts an influence on dogs’ activity, and consequently influences the probability of finding a dog in a given capture effort.

In our study, even with the vehicles driving around in all the streets of the target regions, there was a large number of animals present but not visualized in all captures. Our observations indicate that individual counts based on a census do not adequately estimate the abundance of unrestricted dogs and that the majority of the estimates available in the literature show important biases. Different studies aimed at estimating the abundance of free-roaming dogs did not model or even consider the existence of differences in the probabilities of dog detection [28]. As pointed out before [28], counting techniques should be carried out only in short periods of time and when no other alternative becomes available considering logistics, geography and culture of the study region. Values of capture probabilities obtained in the present study are similar to those estimated by Kalati in a population of urban free-ranging dogs in Kathmandu [85] and may be used as correction factors for the previously published estimates of abundance.

In addition to the limitations already mentioned regarding the observation and sampling techniques applied in capture and recapture studies, issues related to the choice of appropriate analytical methodology deserve mentioning. Environmental and individual variables, relevant to helping understand the population dynamics [30] were not included in our models. The logistics of fieldwork turned out to be complex and difficult, requiring the participation of at least six individuals in each capture effort and extensive fieldwork journeys. Direct contact with animals was unavoidable since assessing the effectiveness of sterilization was one of the study objectives. Studies assessing the population dynamics of dogs, however, could rely on only photographic methods, these being less complex and onerous [86, 87].

Our choice of modeling procedures for open populations allowed for the estimation of survival and recruitment probabilities of unrestricted dogs. In addition, we tested the data for the statistical assumptions required by each model. Model selection followed the AIC technique, which compares favorably with the classic statistical and hypothesis testing [37, 88–90]. Lastly, our parameter estimates and confidence intervals express more faithfully the sources of uncertainty present in the whole estimation process, due to the use of the “model averaging” technique.

The analytical procedures adopted here addressed methodological limitations of previous publications and propose a new starting point for future studies. In our view, longer periods of observation, larger sample sizes, and the choice of more variable study settings including different social, cultural and geographic characteristics are important topics that need the attention of researchers in the field of unrestricted dogs’ ecology. The agenda of the investigation of factors influencing the canine population dynamics must consider the variables addressed in the present study, and further consider the stratification of these population parameters by age groups, as well as by intrinsic animal features and environmental conditions not yet investigated.

## Conclusion

Our estimates of population size in the studied regions in general were small compared to previous estimates in the literature. Survival probability was small and probability of animal entry in the population was high during the 14 months period of follow-up. High turnover, attributed mostly to the abandonment of pet dogs, has important implications to the population composition and the control of zoonosis. Estimates of survival, recruitment and capture probabilities varied over time. Survival and recruitment showed an increasing tendency. Mortality patterns did not differ between genders. The probability of entry in the population was higher among males. The observed population dynamics seem to be driven by density-independent factors. Sterilization, in turn, had no influence upon the parameters analyzed. Our observations are useful for a better understanding of the population dynamics of free-roaming dogs and may aid in the planning, designing and evaluation of population control actions. In this context, it becomes imperative that public health services and nongovernmental organizations develop educational training programs addressing responsible animal ownership and better strategies against abandonment practices. Parameter estimates may also be used as input to new predictive mathematical models. Even though our study generated important answers and new hypotheses, the scarcity of existent knowledge and the misuse of the proper methodology raise numerous relevant questions yet to be elucidated about the population dynamics of free-roaming dogs.

## Supporting information

S1 Appendix Protocol for sterilization and animal recovering procedures. (PDF)

S2 Appendix Informative materials. (PDF)

S3 Appendix Models built. (PDF)

S4 Appendix Individual histories of captures. (PDF)

S5 Appendix Weights of variables. (PDF)
