# Supplementary material for: Abundance, survival, recruitment and effectiveness of sterilization of free-roaming dogs: A capture and recapture study in Brazil
Source: PLoS One. 2017 Nov 1;12(11):e0187233. doi: 10.1371/journal.pone.0187233 (PMC5665538; doi:10.1371/journal.pone.0187233)
Supplement: S1 Appendix — (PDF) [file pone.0187233.s001.pdf]

S1 Appendix: Protocol for sterilization and animal recovering procedures

Sterilization: sterilization of animals testing negative for leishmaniosis followed the analgesic and anaesthetic protocol routinely used by CREVISA. Pre-anaesthetic medication included acepromazine 1%, 0.1mg/kg, tramadol, 1.0 mg/kg and enrofloxacin 10%, 5mg/kg, all by intramuscular route. Anaesthetic medication was administered intravenously 10 minutes after (diazepam 0.5 mg/kg and ketamine 10mg/kg). Epidural anaesthesia using 7 mg/kg of lidocaine was applied on the lumbosacral space (L7-S1) of animals.

Recovering procedures: animals were kept in the CREVISA dependencies for an average period of seven days after surgery. Every twelve hours, dogs received, orally, one dose of enrofloxacin 5.0 mg/kg. In the first three days, every eight hours, dipyrone was administered, 25 mg/kg, orally. Animals received proper cleaning, feeding and dressing. Topic iodopovidone was administered for complimentary operatory asepsis.
