# Supplementary material for: Abundance, survival, recruitment and effectiveness of sterilization of free-roaming dogs: A capture and recapture study in Brazil
Source: PLoS One. 2017 Nov 1;12(11):e0187233. doi: 10.1371/journal.pone.0187233 (PMC5665538; doi:10.1371/journal.pone.0187233)
Supplement: S2 Appendix — (PDF) [file pone.0187233.s002.pdf]

S2 appendix: Informative materials

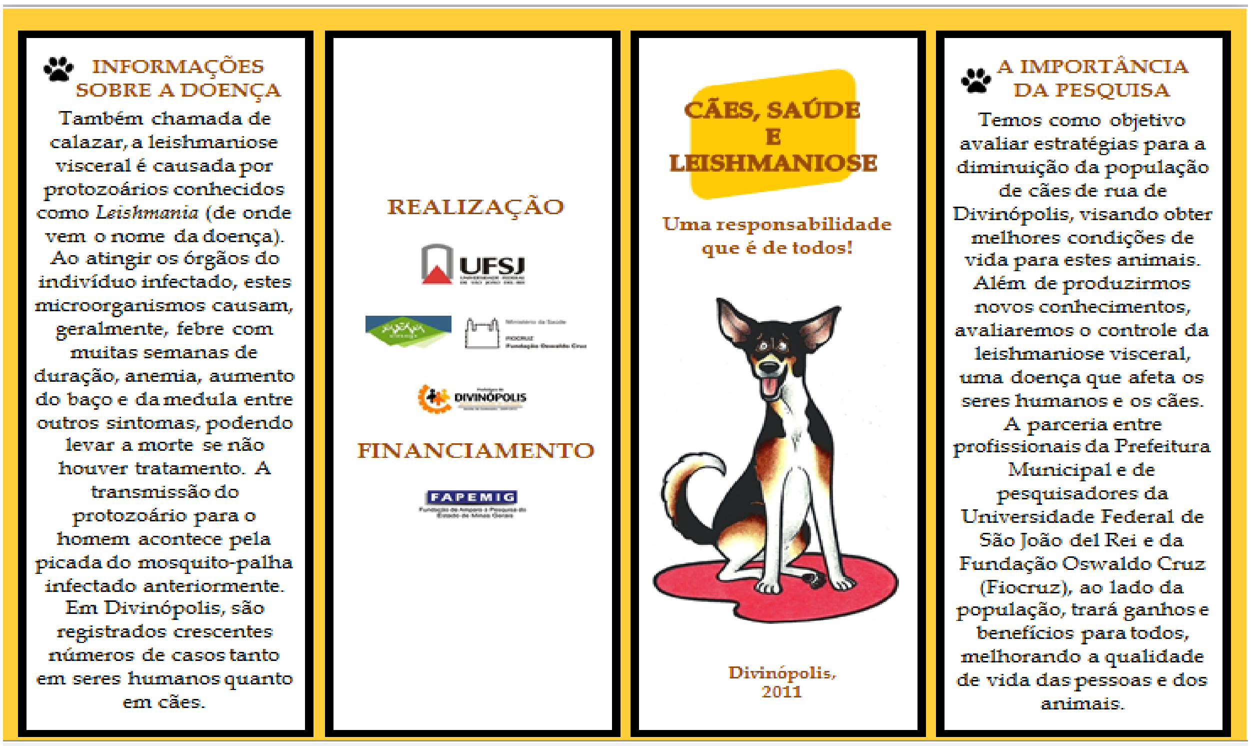

INFORMAÇÕES SOBRE A DOENÇA

Também chamada de calazar, a leishmaniose visceral é causada por protozoários conhecidos como *Leishmania* (de onde vem o nome da doença). Ao atingir os órgãos do indivíduo infectado, estes microorganismos causam, geralmente, febre com muitas semanas de duração, anemia, aumento do baço e da medula entre outros sintomas, podendo levar a morte se não houver tratamento. A transmissão do protozoário para o homem acontece pela picada do mosquito-palha infectado anteriormente. Em Divinópolis, são registrados crescentes números de casos tanto em seres humanos quanto em cães.

REALIZAÇÃO

FINANCIAMENTO

CÃES, SAÚDE E LEISHMANIOSE

Uma responsabilidade que é de todos!

Divinópolis, 2011

A IMPORTÂNCIA DA PESQUISA

Temos como objetivo avaliar estratégias para a diminuição da população de cães de rua de Divinópolis, visando obter melhores condições de vida para estes animais. Além de produzirmos novos conhecimentos, avaliaremos o controle da leishmaniose visceral, uma doença que afeta os seres humanos e os cães. A parceria entre profissionais da Prefeitura Municipal e de pesquisadores da Universidade Federal de São João del Rei e da Fundação Oswaldo Cruz (Fiocruz), ao lado da população, trará ganhos e benefícios para todos, melhorando a qualidade de vida das pessoas e dos animais.

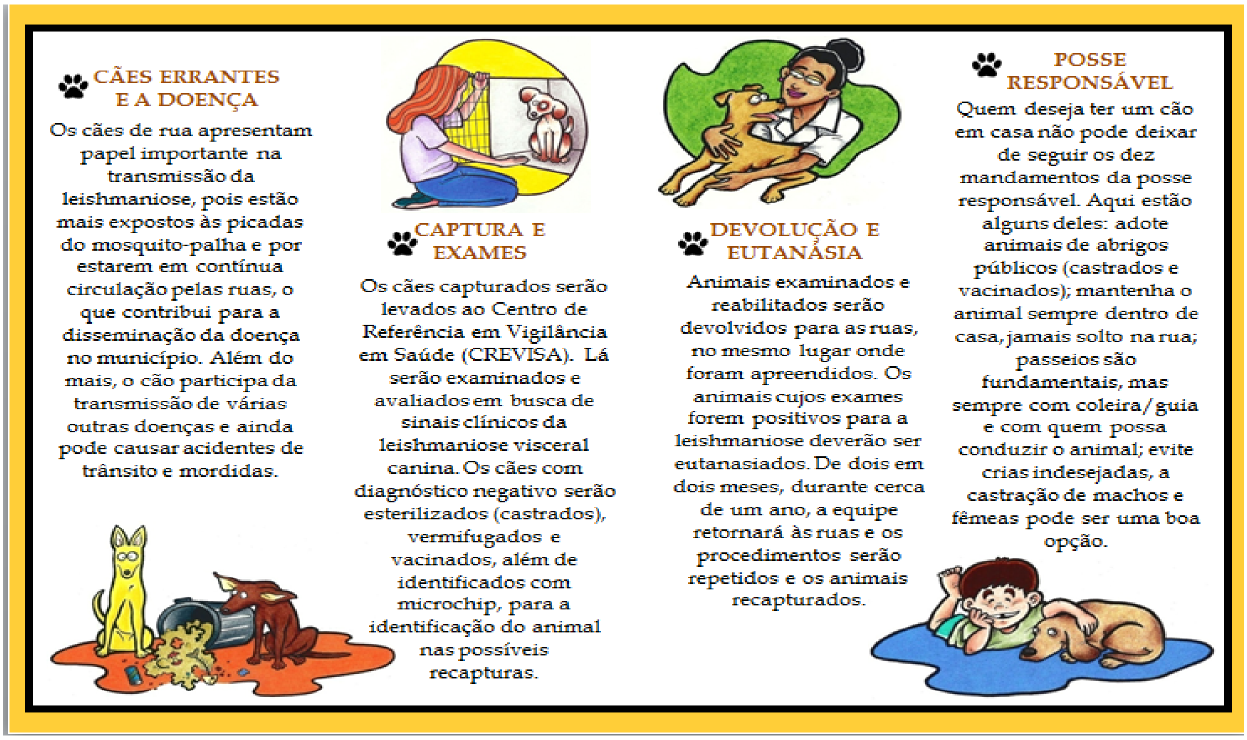

CÃES ERRANTES E A DOENÇA

Os cães de rua apresentam papel importante na transmissão da leishmaniose, pois estão mais expostos às picadas do mosquito-palha e por estarem em contínua circulação pelas ruas, o que contribui para a disseminação da doença no município. Além do mais, o cão participa da transmissão de várias outras doenças e ainda pode causar acidentes de trânsito e mordidas.

CAPTURA E EXAMES

Os cães capturados serão levados ao Centro de Referência em Vigilância em Saúde (CREVISA). Lá serão examinados e avaliados em busca de sinais clínicos da leishmaniose visceral canina. Os cães com diagnóstico negativo serão esterilizados (castrados), vermifugados e vacinados, além de identificados com microchip, para a identificação do animal nas possíveis recapturas.

DEVOLUÇÃO E EUTANÁSIA

Animais examinados e reabilitados serão devolvidos para as ruas, no mesmo lugar onde foram apreendidos. Os animais cujos exames forem positivos para a leishmaniose deverão ser eutanasiados. De dois em dois meses, durante cerca de um ano, a equipe retornará às ruas e os procedimentos serão repetidos e os animais recapturados.

POSSE RESPONSÁVEL

Quem deseja ter um cão em casa não pode deixar de seguir os dez mandamentos da posse responsável. Aqui estão alguns deles: adote animais de abrigos públicos (castrados e vacinados); mantenha o animal sempre dentro de casa, jamais solto na rua; passeios são fundamentais, mas sempre com coleira/guia e com quem possa conduzir o animal; evite crias indesejadas, a castração de machos e fêmeas pode ser uma boa opção.
