# Supplementary material for: Abundance, survival, recruitment and effectiveness of sterilization of free-roaming dogs: A capture and recapture study in Brazil
Source: PLoS One. 2017 Nov 1;12(11):e0187233. doi: 10.1371/journal.pone.0187233 (PMC5665538; doi:10.1371/journal.pone.0187233)
Supplement: S3 Appendix — (PDF) [file pone.0187233.s003.pdf]

S3 appendix: Models built

➔ **Gender***

Model	QAICc	Delta QAICc	AICc Weights	Number of parameters
$\Phi_t\ p_t\ b_t$	1129,107	0	0,73242	20
$\Phi_{g+t}\ p_t\ b_t$	1132,845	3,7376	0,11302	22
$\Phi_t\ p_{g+t}\ b_t$	1133,357	4,2491	0,08751	22
$\Phi_{g+t}\ p_{g+t}\ b_t$	1134,752	5,645	0,04355	23
$\Phi_{g*t}\ p_t\ b_t$	1137,084	7,9762	0,01358	25
$\Phi_t\ p_{g*t}\ b_t$	1139,176	10,0684	0,00477	26
$\Phi_{g+t}\ p_t\ b_{g*t}$)	1141,136	12,0286	0,00179	28
$\Phi_t\ p_{g+t}\ b_{g*t}$	1141,612	12,505	0,00141	28
$\Phi_{g*t}\ p_{g+t}\ b_t$	1143,472	14,3645	0,00056	28
$\Phi.\ p_t\ b_t$	1143,805	14,6976	0,00047	16
$\Phi_{g+t}\ p_{g*t}\ b_t$	1144,995	15,8878	0,00026	29
$\Phi_g\ p_{g+t}\ b_t$	1145,361	16,2533	0,00022	17
$\Phi_g\ P_t\ b_t$	1145,587	16,4792	0,00019	17
$\Phi.\ p_{g+t}\ b_t$	1145,922	16,8148	0,00016	17
$\Phi_t\ p_{g*t}\ b_{g*t}$	1148,406	19,2982	0,00005	32
$\Phi_{g*t}\ p_{g+t}\ b_{g*t}$	1151,58	22,4721	0,00001	34
$\Phi.\ p_t\ b_{g*t}$	1151,868	22,7606	0,00001	22
$\Phi_g\ p_t\ b_{g*t}$	1153,369	24,2619	0	23
$\Phi_{g+t}\ p_{g*t}\ b_{g*t}$	1153,571	24,4634	0	35
$\Phi_g\ p_{g+t}\ b_{g*t}$	1153,571	24,4636	0	24
$\Phi.\ p_{g+t}\ b_{g*t}$	1153,681	24,5734	0	23
$\Phi_{g*t}\ p_{g*t}\ b_{g*t}$	1154,353	25,246	0	36
$\Phi_t\ p_t\ b_{g*t}$	1154,437	25,3294	0	26
$\Phi_{g+t}\ p_{g+t}\ b_{g*t}$	1155,779	26,6715	0	35
$\Phi.\ p_{g*t}\ b_t$	1155,848	26,7403	0	23
$\Phi_g\ p_{g*t}\ b_t$	1157,344	28,2367	0	24
$\Phi_{g*t}\ p_{g*t}\ b_t$	1158,256	29,1482	0	32
$\Phi_{p*t}\ p_t\ b_{g*t}$	1161,664	32,5568	0	31
$\Phi_g\ p_{g*t}\ b_{g*t}$	1164,145	35,0371	0	30
$\Phi.\ p_{g*t}\ b_{g*t}$	1164,58	35,4726	0	29
$\Phi_t\ p_g\ b_t$	1166,592	37,4846	0	15
$\Phi_t\ p.\ b_t$	1166,609	37,5015	0	15
$\Phi_{g+t}\ p.\ b_t$	1168,331	39,2239	0	16
$\Phi_{g+t}\ p_g\ b_t$	1170,097	40,9893	0	17
$\Phi_t\ p_g\ b_{g*t}$	1174,979	45,8718	0	21
$\Phi_{g+t}\ p_g\ b_{g*t}$	1175,076	45,9689	0	22
$\Phi_t\ p.\ b_{g*t}$	1175,14	46,0323	0	21
$\Phi.\ p.\ b_t$	1176,024	46,9168	0	10

Φ_{g+t} p. b_{g*t}	1176,602	47,4941	0	22
Φ_{g*t} p_g b_t	1176,791	47,6835	0	21
Φ_{g*t} p. b_t	1176,791	47,6835	0	21
Φ_g p. b_t	1177,615	48,5074	0	11
Φ. p_g b_t	1177,756	48,6483	0	11
Φ_g p_g b_t	1178,408	49,3009	0	12
Φ_{g*t} p_g b_{g*t}	1182,364	53,257	0	28
Φ. p. b_{g*t}	1183,79	54,6823	0	16
Φ_g p_g b_{g*t}	1184,727	55,6199	0	18
Φ. p_g b_{g*t}	1185,332	56,2241	0	17
Φ_g p. b_{g*t}	1185,348	56,2407	0	17
Φ_{g*t} p. b_{g*t}	1186,034	56,9267	0	27

* Φ= survival; p= probability of capture; b= probability of entry; t= parameter values vary in different capture occasions; g+t= additive model in which parameter values varied with time and gender; g*t= interaction between gender and time; **.** (dot)= parameter value constant in all captures; g**.**= parameter values constants, but different in males and females.

➔ **Area****

**Model**	**QAICc**	**Delta QAICc**	**AICc Weights**	**Number of parameters**
Φ_t p_t b_t	1067,566	0	0,57923	20
Φ_{g+t} p_t b_t	1069,836	2,27	0,18618	22
Φ_{g+t} p_{g+t} b_t	1070,334	2,7679	0,14515	23
Φ. p_{g+t} b_t	1073,371	5,8056	0,03178	17
Φ_t p_{g*t} b_t	1073,547	5,9814	0,02911	26
Φ_{g*t} p_t b_t	1075,453	7,8876	0,01122	25
Φ_t p_t b_{g*t}	1076,924	9,3585	0,00538	26
Φ_t p_{g+t} b_{g*t}	1078,287	10,7215	0,00272	28
Φ_{g+t} p_t b_{g*t}	1079,043	11,4771	0,00186	28
Φ_{g+t} p_{g*t} b_t	1079,262	11,6962	0,00167	29
Φ_{g+t} p_{g+t} b_{g*t}	1079,513	11,9476	0,00147	29
Φ_{g*t} p_{g+t} b_t	1080,448	12,8826	0,00092	28
Φ_g p_t b_t	1080,533	12,9669	0,00089	17
Φ. p_t b_t	1080,611	13,0457	0,00085	16
Φ_g p_{g+t} b_t	1081,415	13,8496	0,00057	18
Φ_t p_{g+t} b_t	1082,279	14,713	0,00037	28
Φ_t p_{g*t} b_{g*t}	1082,727	15,1617	0,0003	32
Φ_{g*t} p_{g*t} b_{g*t}	1084,59	17,024	0,00012	31
Φ_g p_{g+t} b_{g*t}	1086,112	18,5461	0,00005	23
Φ. P_{g+t} b_{g*t}	1086,288	18,7223	0,00005	23
Φ_g p_t b_{g*t}	1087,38	19,8142	0,00003	23
Φ. p_t b_{g*t}	1087,443	19,8776	0,00003	22
Φ. P_{g*t} b_t	1088,486	20,9204	0,00002	23
Φ_{g+t} p_{g*t} b_{g*t}	1088,517	20,9513	0,00002	35

$\Phi_{g*t}\ p_{g+t}\ b_{g*t}$	1089,659	22,0933	0,00001	34
$\Phi_{g}\ p_{g*t}\ b_{t}$	1090,012	22,446	0,00001	24
$\Phi_{g*t}\ p_{g*t}\ b_{g*t}$	1091,975	24,4091	0	38
$\Phi_{.}\ p_{g*t}\ b_{g*t}$	1096,247	28,6813	0	29
$\Phi_{g}\ p_{g*t}\ b_{g*t}$	1098,02	30,4543	0	30
$\Phi_{t}\ p_{g}\ b_{t}$	1098,034	30,4678	0	16
$\Phi_{g*t}\ p_{g*t}\ b_{t}$	1098,444	30,8778	0	32
$\Phi_{g+t}\ p_{.}\ b_{t}$	1100,307	32,7416	0	15
$\Phi_{g+t}\ p_{g}\ b_{t}$	1100,408	32,8425	0	17
$\Phi_{t}\ p_{.}\ b_{t}$	1101,877	34,3116	0	15
$\Phi_{.}\ p_{g}\ b_{t}$	1106,769	39,2035	0	11
$\Phi_{t}\ p_{g}\ b_{g*t}$	1106,998	39,4319	0	22
$\Phi_{g}\ p_{g}\ b_{t}$	1108,847	41,2817	0	12
$\Phi_{g+t}\ p_{g}\ b_{g*t}$	1109,32	41,7544	0	23
$\Phi_{g*t}\ p_{g}\ b_{t}$	1109,574	42,0082	0	22
$\Phi_{t}\ p_{.}\ b_{g*t}$	1110,007	42,4412	0	21
$\Phi_{.}\ p_{.}\ b_{t}$	1110,016	42,4502	0	10
$\Phi_{g}\ p_{.}\ b_{t}$	1110,165	42,5996	0	11
$\Phi_{g+t}\ p_{.}\ b_{g*t}$	1110,215	42,6497	0	22
$\Phi_{g*t}\ p_{.}\ b_{t}$	1112,215	44,6489	0	21
$\Phi_{.}\ P_{g}\ b_{g*t}$	1115,106	47,5399	0	17
$\Phi_{g}\ p_{.}\ b_{g*t}$	1115,99	48,4239	0	17
$\Phi_{.}\ P_{g*t}\ b_{g*t}$	1116,55	48,9843	0	16
$\Phi_{g}\ p_{g}\ b_{g*t}$	1116,998	49,4319	0	18
$\Phi_{g*t}\ p_{g}\ b_{g*t}$	1120,661	53,0957	0	27
$\Phi_{g*t}\ p_{t}\ b_{g*t}$	1120,661	53,0957	0	27

** Φ= survival; p= probability of capture; b= probability of entry; t= parameter values vary in different capture occasions; g+t= additive model in which parameter values varied with time and gender; g*t= interaction between gender and time; **.** (dot)= parameter value constant in all captures; g**.**= parameter values constants, but different in áreas A and B.
