# Supplementary material for: Abundance, survival, recruitment and effectiveness of sterilization of free-roaming dogs: A capture and recapture study in Brazil
Source: PLoS One. 2017 Nov 1;12(11):e0187233. doi: 10.1371/journal.pone.0187233 (PMC5665538; doi:10.1371/journal.pone.0187233)
Supplement: S4 Appendix — (PDF) [file pone.0187233.s004.pdf]

S4 appendix: Individual histories of captures

➔ **Gender**

History*	F**	M**
0000001	9	22;
0000001	-1***	0;
0000010	5	16;
0000010	-1	0;
0000011	3	5;
0000100	7	13;
0000100	-2	-1;
0000101	1	3;
0000110	3	3;
0000111	0	2;
0001000	5	9;
0001000	-1	-4;
0001001	3	3;
0001010	1	1;
0001011	0	1;
0001100	1	2;
0001101	0	1;
0001110	0	1;
0001111	0	1;
0010000	14	6;
0010000	0	-2;
0010001	1	2;
0010010	1	2;

0010100	1	2;
0010011	0	1;
0010110	1	0;
0011000	0	1;
0011010	0	1;
0011100	0	1;
0100000	11	18;
0100000	-1	-3;
0100010	1	0;
0100100	1	0;
0100110	1	0;
0101000	3	2;
0101001	1	0;
0101010	0	1;
0101101	1	1;
0110000	3	1;
0110001	1	0;
0110010	0	1;
0110110	1	0;
0111000	1	2;
0111111	0	1;
1000000	12	21;
1000000	-5	-5;
1001000	1	3;
1000001	1	0;
1001010	0	2;
1010000	2	5;

1010001	1	2;
1010101	1	0;
1010110	0	1;
1011011	1	0;
1100000	4	9;
1100001	1	0;
1100010	1	0;
1100011	1	1;
1100100	2	2;
1101000	0	1;
1101001	1	0;
1101100	0	1;
1101010	0	1;
1101011	1	0;
1101101	0	1;
1110000	0	5;
1110100	1	0;
1110110	0	1;
1111000	3	1;
1111010	1	1;
1111101	1	0;
1111110	1	1;
1111111	2	2;

*** 0=animal not captured (or no viewed) / 1=animal captured (or viewed)**

**** F=female; M=male (number of dogs with the history of captures described - stratified by gender)**

***** Negative sign indicates the occurrence of death during the capture procedure.**

➔ **Area**

History*	A**	B
0000001	18	13;
0000001	-1***	0;
0000010	10	11;
0000010	-1	0;
0000011	3	5;
0000100	14	6;
0000100	-1	-2;
0000101	2	2;
0000110	5	1;
0000111	2	0;
0001000	8	6;
0001000	-1	-4;
0001001	3	3;
0001010	2	0;
0001011	0	1;
0001100	1	2;
0001101	1	0;
0001110	0	1;
0001111	0	1;
0010000	11	9;
0010000	-1	-1;
0010001	3	0;
0010010	1	2;

0010100	2	1;
0010011	0	1;
0010110	1	0;
0011000	0	1;
0011010	1	0;
0011100	1	0;
0100000	16	13;
0100000	-2	-2;
0100010	1	0;
0100100	1	1;
0100110	1	0;
0101000	4	1;
0101001	1	0;
0101010	1	0;
0101101	2	0;
0110000	2	2;
0110001	1	0;
0110010	0	1;
0110110	0	1;
0111000	0	3;
0111111	0	1;
1000000	19	14;
1000000	-3	-7;
1001000	4	0;
1000001	0	1;

1001010	0	2;
1010000	4	3;
1010001	0	3;
1010101	1	0;
1010110	0	1;
1011011	0	1;
1100000	6	7;
1100001	0	1;
1100010	1	0;
1100011	1	1;
1100100	2	2;
1101000	1	0;
1101001	1	0;
1101100	1	0;
1101010	0	1;
1101011	0	1;
1101101	0	1;
1110000	4	1;
1110100	0	1;
1110110	0	1;
1111000	1	3;
1111010	0	2;
1111101	0	1;
1111110	0	2;
1111111	2	2;

*** 0=animal not captured (or not viewed) / 1=animal captured (or viewed)**

**** A=Area A (control); B=area B (intervention) (number of dogs with the history of captures described - stratified by area)**

***** Negative sign indicates the occurrence of death during the capture procedure.**
