# Supplementary material for: Abundance, survival, recruitment and effectiveness of sterilization of free-roaming dogs: A capture and recapture study in Brazil
Source: PLoS One. 2017 Nov 1;12(11):e0187233. doi: 10.1371/journal.pone.0187233 (PMC5665538; doi:10.1371/journal.pone.0187233)
Supplement: S5 Appendix — (PDF) [file pone.0187233.s005.pdf]

S5 appendix: Weights of variables

$\mathbf{\Phi_t}$ = 0.819933	$\mathbf{p_t}$ = 0.85902	$\mathbf{b_t}$ = 0.99008
$\mathbf{\Phi_{g+t}}$ = 0.15657	$\mathbf{p_{g+t}}$ = 0.13106	$\mathbf{b_{g*t}}$ = 0
$\mathbf{\Phi_{g*t}}$ = 0.01358	$\mathbf{p_{g*t}}$ = 0	
**Φ.**(no variation) = 0	**p.** (no variation) = 0	

1. Variable weights given by the set of models containing “gender”

$\mathbf{\Phi_t}$ = 0,60834	$\mathbf{p_t}$ = 0.77663	$\mathbf{b_t}$ = 0.98267
$\mathbf{\Phi_{a+t}}$ = 0.33133	$\mathbf{p_{a+t}}$ = 0.17693	$\mathbf{b_{a*t}}$ = 0
**Φ.** (sem variação) = 0.03178	$\mathbf{p_{a*t}}$ = 0.02911	
$\mathbf{\Phi_{a*t}}$ = 0.01122	**p.** = 0	

2. Variable weights given by the set of models containing "area"
